# Spontaneous Medial Rectus Haematoma: A Sight-Threatening Complication of Warfarin Toxicity

**DOI:** 10.7759/cureus.77995

**Published:** 2025-01-26

**Authors:** Sara Sharif, Haider Shah, Paras Agarwal

**Affiliations:** 1 Ophthalmology, University of Liverpool, Liverpool, GBR; 2 Opthalmology, Arrowe Park Hospital, Birkenhead, GBR; 3 Ophthalmology, Warrington Hospital, Warrington and Halton Hospitals NHS Foundation Trust, Warrington, GBR

**Keywords:** canthotomy, ocular proptosis, orbit, orbital haemorrhage, ‏strabismus, warfarin toxicity

## Abstract

This report highlights a rare instance of spontaneous medial rectus haematoma in a 77-year-old female, attributed to warfarin use, underscoring its clinical significance. The patient initially presented with acute right eye pain, peri-orbital swelling, and reduced vision. Examination revealed a tense globe and elevated intraocular pressure (IOP), prompting emergency lateral canthotomy and inferior cantholysis. However, despite the initial procedure, her IOP remained elevated, indicating direct pressure from the haematoma on the globe and necessitating additional upper and lower eyelid lateral cantholysis. Medical management was also initiated to control the IOP. Over four months, the patient's visual acuity improved significantly, although a relative afferent pupillary defect persisted. This case underscores the rarity of spontaneous medial rectus haematoma in anticoagulated patients and highlights the critical importance of vigilant international normalized ratio (INR) monitoring and timely intervention to reduce morbidity and the risk of vision loss.

## Introduction

Intra-orbital haemorrhage is a rare but sight-threatening condition, most commonly associated with trauma [1]. The accumulation of blood within the rigid orbital cavity can lead to acute orbital compartment syndrome, necessitating urgent intervention to prevent vision loss [1]. Non-traumatic aetiologies include connective tissue disorders, bleeding diatheses, or activities that increase intrathoracic pressure, such as Valsalva manoeuvres [2]. Spontaneous retro-bulbar haemorrhages associated with warfarin or direct oral anticoagulants (DOACs) have been reported, albeit extremely rare [3,4]. To our knowledge, this is the first documented case of a spontaneous haemorrhage localised within the medial rectus muscle, attributed to warfarin toxicity.

## Case presentation

A 77-year-old female patient initially presented to the emergency eye clinic with an acute spontaneous sub-conjunctival haemorrhage. Over the following two days, her symptoms worsened, and she returned with right eye pain, peri-orbital swelling, and reduced vision. She denied any history of coughing, vomiting, or preceding trauma and reported being medically stable prior to the onset of symptoms.

Significant medical history included multiple unprovoked pulmonary emboli, chronic kidney disease (CKD) with a failed renal transplant requiring haemodialysis, gastric ulcers, and ischaemic heart disease treated with coronary stents. Additionally, she had a history of endometrial cancer that was initially treated surgically but recurred 14 years later. As she was not a candidate for further surgery, treatment with medroxyprogesterone acetate (Provera®) was initiated. Due to her increased thromboembolic risk, she had been started on warfarin several years prior.

On examination, Snellen visual acuity was hand movements in the right eye and 6/9 in the left eye. Examination revealed a swollen, tense, right peri-orbital region with associated ecchymosis. Hyper- and exo-deviation of the right eye were noted. An extensive 360-degree sub-conjunctival haemorrhage involved the anterior globe, obscuring the view of the cornea. IOP, measured with an iCare IC100 tonometer (Icare Finland Oy, Vantaa, Finland), was 40 mmHg. A relative afferent pupillary defect (RAPD) was suspected; however, due to limited visualisation of the cornea, a more detailed assessment was not feasible.

Given the tense globe, compromised vision, and significantly elevated IOP, an emergency lateral canthotomy and inferior cantholysis were performed, leading to partial alleviation of her symptoms.

**Figure 1:**
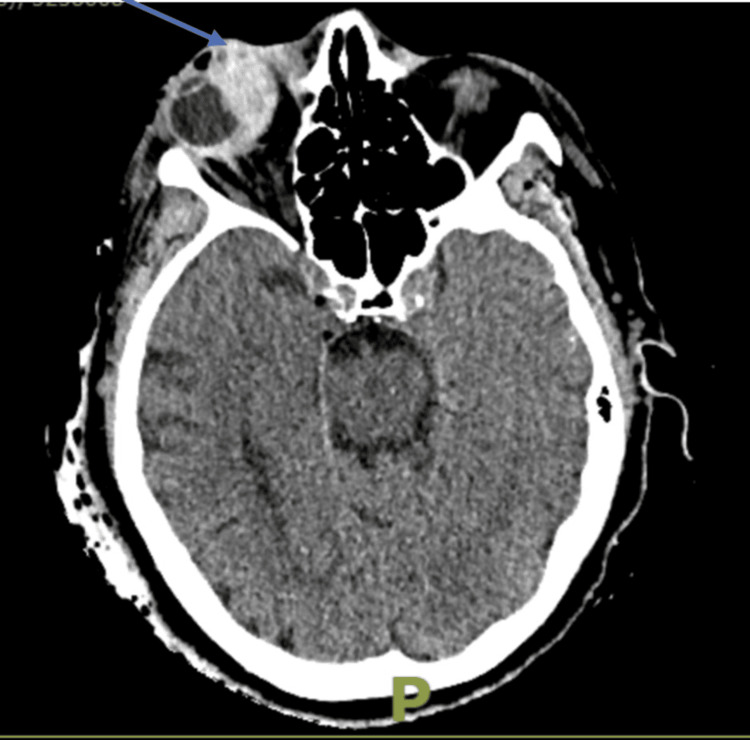
CT of brain and orbits showing a large right sided medial rectus haematoma (blue arrow) extending into the intraconal space.

Orbital computed tomography (CT) imaging of her brain and orbits revealed an isolated haematoma within the right medial rectus muscle, accompanied by extensive sub-conjunctival haemorrhage extending into the intraconal space (Figure 1). The paranasal sinuses were clear, and there was no evidence of orbital fracture. The patient’s international normalized ratio (INR) at presentation was 3 (therapeutic range: 2-3), prompting the suspension of warfarin therapy.

The following day, the patient attended her routine dialysis appointment and was observed to have worsening periorbital pain and swelling, causing new-onset agitation and confusion. Examination revealed a recurrence of orbital tension with no laxity of the eyelids. The globe remained obscured by sub-conjunctival haemorrhage and chemosis, with early breakdown of the nasal conjunctiva and overlying clot formation. The eye was hyper- and exo-deviated, with superotemporal displacement of the cornea beneath the upper eyelid.

An extended lateral canthotomy with repeated lower eyelid cantholysis, along with additional upper eyelid cantholysis, was performed. Verbal consent was obtained from her husband due to the patient’s lack of capacity. Lidocaine 1% (4 mL) was infiltrated into the lateral canthal region, although it provided limited anaesthetic effect. St. Martin forceps and Westcott scissors were utilised during the procedure. Orbital tension was relieved three minutes post procedure and laxity of the upper and lower eyelids was confirmed. Owing to persistent agitation, the patient was transferred to the intensive therapy unit (ITU) for closer monitoring.

Post-procedure haemostasis was not required to allow for continued drainage of the haemorrhage, but serial monitoring of haemoglobin (Hb) and INR was initiated. The patient’s Hb decreased from 88 g/L to 75 g/L, accompanied by tachycardia, necessitating the transfusion of one unit of packed red blood cells. Her INR rose to 4.2, prompting the administration of 20 units/kg of human prothrombin complex (Octaplex®).

In light of the significant reduction in Hb and her history of gastric ulcers, a gastroscopy was performed under general anaesthesia, revealing gastritis and a hiatus hernia, but no active bleeding. A more detailed ocular examination was conducted concurrently, demonstrating hyper- and exo-deviation of the globe, extensive 360-degree sub-conjunctival haemorrhage, and nasal conjunctival breakdown with overlying clot, which was cleaned. The globe was no longer tense, with significant laxity in the upper and lower eyelids; however, IOP was recorded at 38 mmHg. The cornea was clear but covered by the upper eyelid, with no anterior segment abnormality. RAPD was observed in the right eye, but no signs of optic disc swelling or pallor were present. Examination of the left eye was unremarkable.

The patient was started on topical Ganfort® eye drops once daily, brimonidine and brinzolamide eye drops twice daily, and chloramphenicol ointment four times daily in the right eye. No further episodes of bleeding were observed, her INR normalised to 1.0, and her Hb stabilised at approximately 90 g/L. The patient’s clinical condition improved, with a resolution of confusion. She was discharged one week after admission.

**Figure 2:**
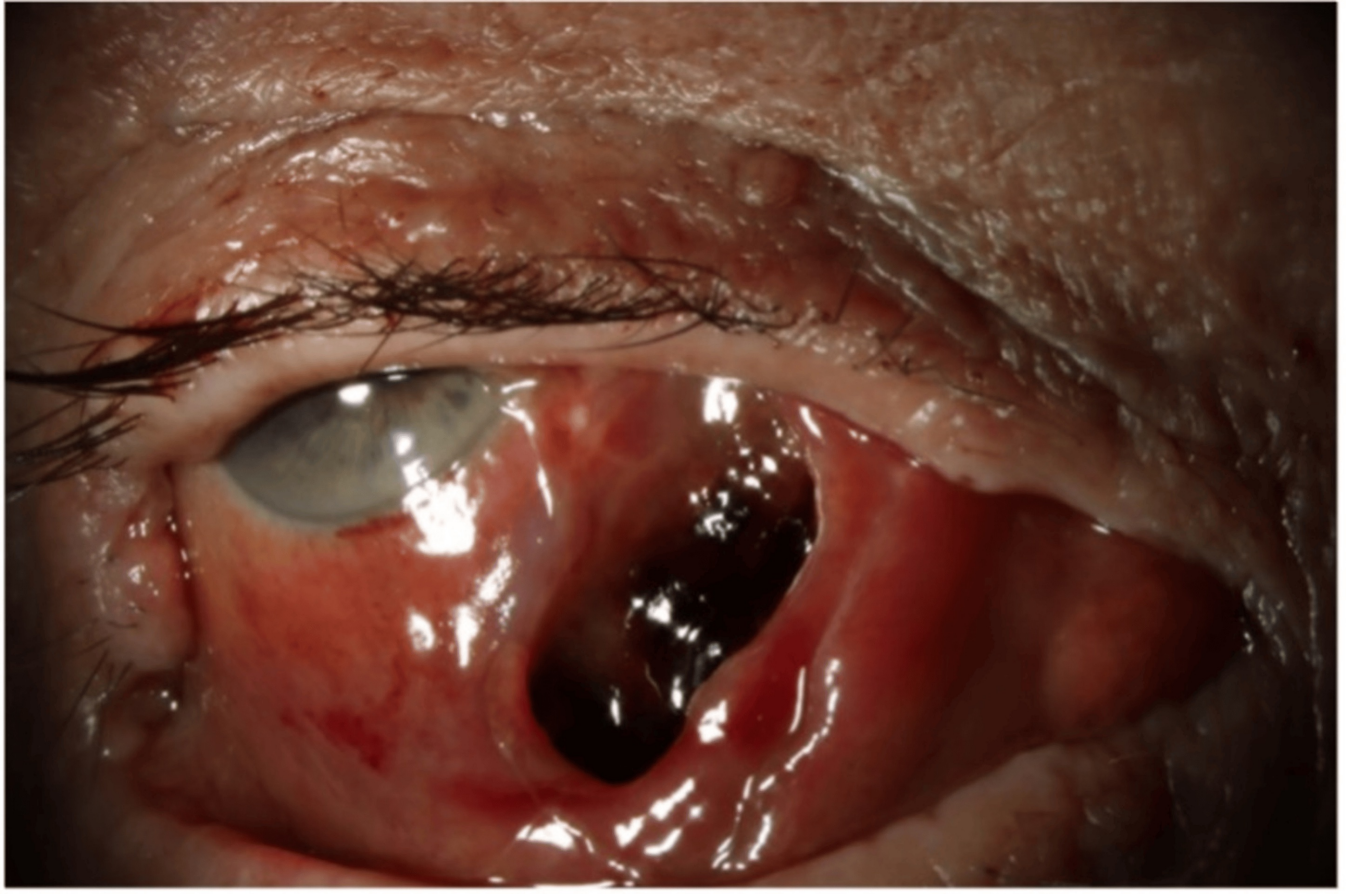
Anterior segment photograph of right eye, 15 days post the onset of haematoma. Extensive sub-conjunctival haemorrhage with large medial rectus haematoma is noted. Eye significantly hyper and exo-deviated. Note: The patient is attempting to adduct the eye in this image.

Ten days post examination under anaesthesia (five days following right eye orbital compartment syndrome), the patient was reviewed in the eye clinic. Visual acuity of the right eye was perception of light with a notable RAPD. However, significant clinical improvement of the sub-conjunctival haemorrhage and medial rectus haematoma was noted. The right eye globe position was unchanged, with no ability to adduct the eye (Figure 2). The IOP had decreased to 7 mmHg. There was significant lid laxity and a non-tense peri-orbital region. The patient was advised to switch to latanoprost eye drops once daily and continue chloramphenicol ointment four times a day, and was started on prednisolone 1% eye drops four times a day in the right eye.

**Figure 3:**
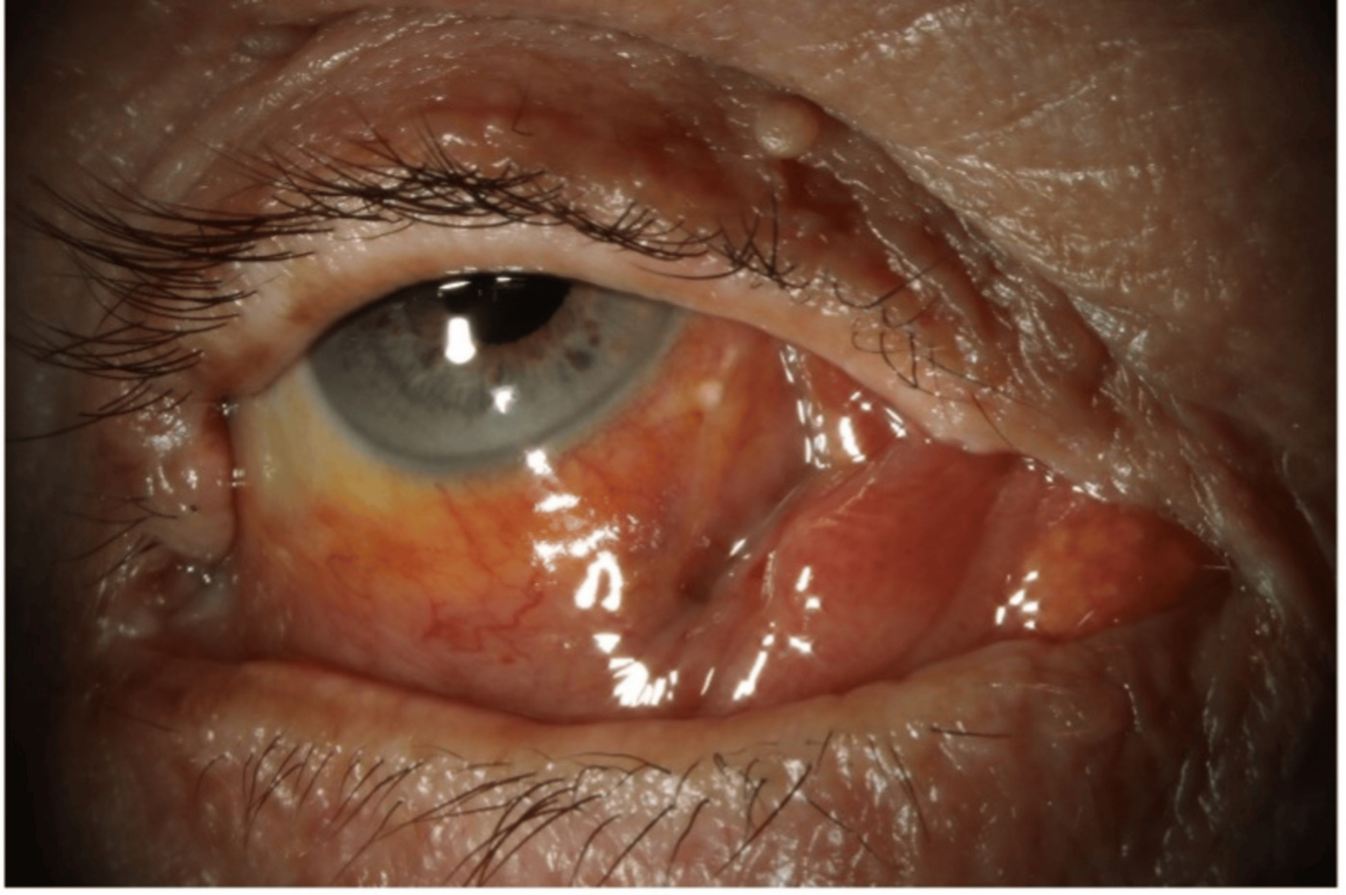
Anterior segment photograph of right eye, 25 days post the onset of haematoma. Note: The patient is attempting to adduct the eye in this image.

A follow-up examination 10 days later demonstrated marked clinical resolution. Snellen visual acuity had improved to 6/18 in the right eye, with improvement of right eye position, reduction of sub-conjunctival swelling, and no visible medial rectus haematoma (Figure 3). As IOP was 9 mmHg, all IOP-lowering drops were suspended, and the patient was instructed to continue the steroid drops and initiate intensive ocular lubricant drops. 

**Figure 4:**
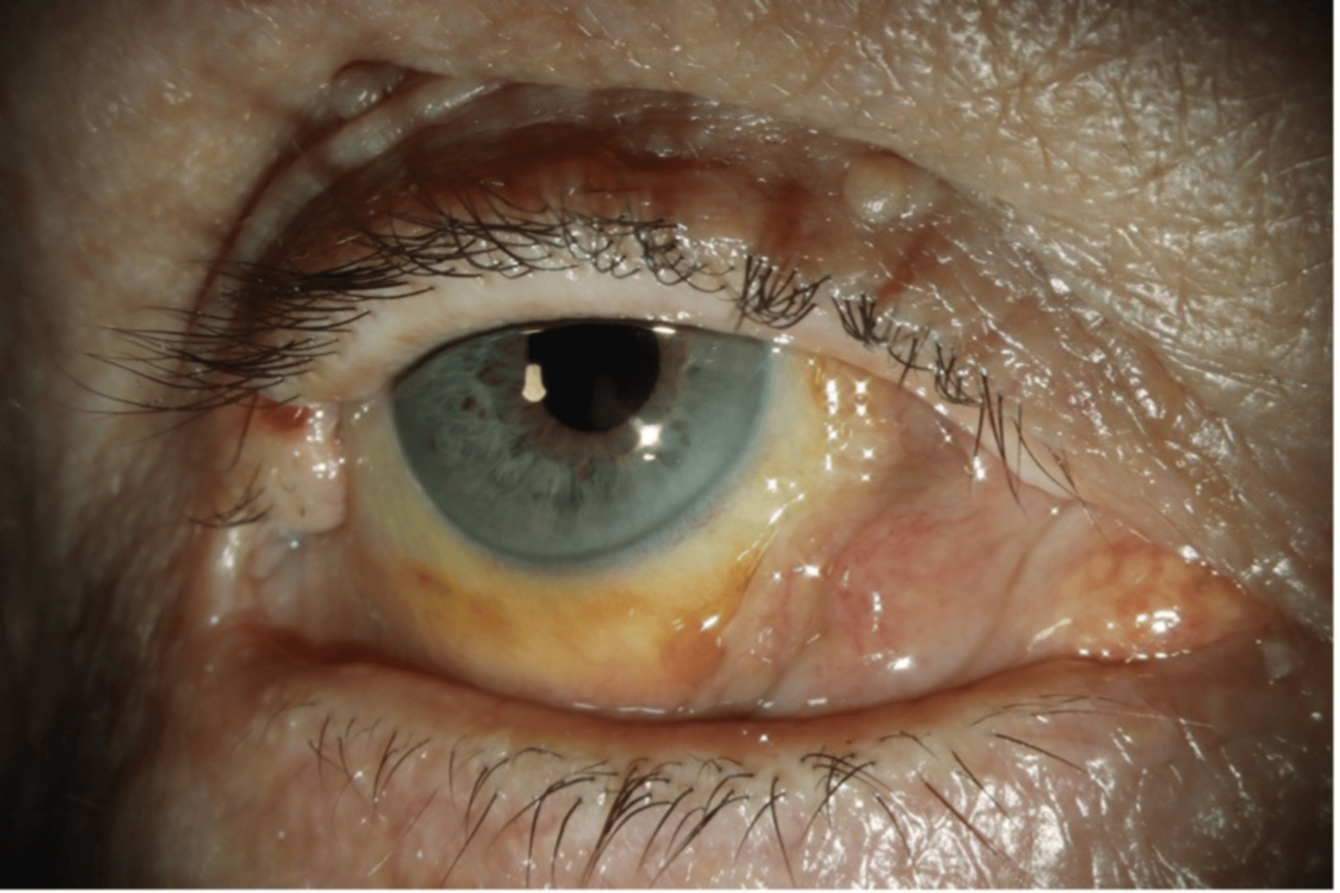
Anterior segment photograph of the right eye, four months post haematoma. Residual nasal conjunctival thickening only.

**Figure 5:**
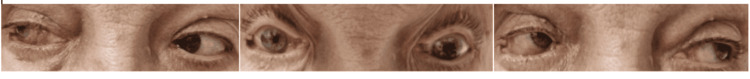
A series of three photographs illustrating horizontal gaze positions, four months post haematoma. Orthotropia in primary position when the lids are lifted, and full adduction of the right eye.

Four months post haematoma, Snellen visual acuity had improved to 6/6 in both eyes, with an unchanged right RAPD. IOP was 10 mmHg in the right eye and 13 mmHg in the left eye. In primary gaze, she had a well-controlled minimal exophoria (1 prism dioptre) with full medial rectus function. On extreme right gaze, the patient noted mild vertical diplopia with a 5 prism dioptre right eye hypertropia. At times, there appeared to be a pseudo-hypertropia due to lid malposition of the right eye. The conjunctiva over the medial rectus remains thickened (Figure 4). A generalised reduction of ganglion cell layer of the right eye was noted on optical coherence topography (OCT) along with a superior arcuate field defect observed on Humphrey Visual Field (HVF) testing. Figure 5 illustrates the patient's horizontal gaze positions.

## Discussion

Orbital haemorrhages are rare but potentially sight-threatening events characterised by bleeding within the orbital cavity, often requiring urgent intervention to prevent vision loss [5]. Spontaneous non-traumatic orbital haemorrhages are even less common, often associated with predisposing vascular abnormalities [6]. Orbital haemorrhages are classified as anterior (e.g., sub-conjunctival) or posterior (e.g., retrobulbar). While spontaneous retrobulbar haemorrhages are documented, spontaneous medial rectus haematomas are exceptionally rare, with limited understanding of their aetiology [7].

The contributing factors include trauma, systemic conditions, or anticoagulation therapy. Symptoms such as pain, proptosis, reduced vision, and raised IOP may result in optic nerve compression and ischaemia, necessitating prompt intervention to prevent morbidity [8]. Imaging with CT or magnetic resonance imaging (MRI) is essential for diagnosis, and surgical procedures such as canthotomy are often required to address orbital compartment syndrome [9].

Warfarin, an anticoagulant that inhibits vitamin K-dependent clotting factors, increases bleeding risk, particularly with elevated INR levels. Despite the patient's INR being within the therapeutic range (3.0), it was at the upper limit, increasing the risk of spontaneous bleeding. The comorbidities contributing to this patient’s susceptibility included recurrent pulmonary emboli, CKD requiring haemodialysis, ischaemic heart disease, and recurrent endometrial cancer [10]. CKD and haemodialysis impair platelet function and vascular health, whereas medroxyprogesterone acetate, prescribed for recurrent cancer, poses additional thromboembolic risk [10]. These factors likely combined to precipitate the spontaneous medial rectus haematoma.

This case aligns with previous reports of orbital haemorrhages in anticoagulated patients, such as bilateral retrobulbar haemorrhage associated with warfarin misuse [11]. These cases underscore the importance of close INR monitoring to prevent spontaneous haemorrhagic complications. DOACs, which carry lower bleeding risks and do not require routine monitoring, offer potential alternatives. Meta-analyses suggest DOACs like apixaban and dabigatran reduce major bleeding risk by 40% and 21%, respectively, compared with warfarin [12].

Differential diagnoses were carefully considered. Intracranial haemorrhage, which is often a concern in anticoagulated patients, was excluded based on imaging. Orbital cellulitis, typically accompanied by fever, malaise, and sinus involvement, was unlikely given the absence of systemic or infective markers [13]. Rapid symptom onset and imaging findings excluded orbital neoplasms and tumours. Finally, carotid-cavernous fistula, characterised by proptosis, arterialised conjunctival vessels, and orbital bruit, was eliminated based on clinical and imaging findings [13]. The clinical presentation and investigations strongly supported a spontaneous medial rectus haematoma as the diagnosis.

Management of orbital haemorrhages, particularly those with orbital compartment syndrome, prioritises preventing vision loss [14]. Prompt assessment, imaging, and ophthalmic evaluation are critical. Signs of orbital compartment syndrome, such as proptosis, raised IOP, and reduced vision, necessitate emergency lateral canthotomy and cantholysis. Anticoagulation therapy should be suspended, with reversal agents administered as needed. Close monitoring of IOP, optic nerve function, and overall recovery ensures the resolution of symptoms [14]. In this case, however, it is important to note that although the IOP improved following adequate lateral canthotomy and cantholysis, it remained significantly elevated. We postulate that this was due to a direct pressure effect from the haematoma and enlarged medial rectus directly upon the globe. Theunte and Neely describe a similar report of spontaneous haemorrhage within the medial rectus of a patient, however, this was associated with pancytopenia secondary to acute myelogenous leukaemia [15]. The present case underscores the importance of considering spontaneous orbital haemorrhage in patients with haematologic malignancies who present with sudden proptosis, even in the absence of trauma.

## Conclusions

Our case highlights the rarity of spontaneous medial rectus haematoma, particularly in the context of warfarin therapy. The patient’s anticoagulation, combined with comorbidities, likely contributed to vessel fragility and haemorrhagic complications. It is important to note that lateral canthotomy and cantholysis may not result in the normalisation of IOP; hence, additional measures may be required. Regular coagulation monitoring, early recognition, and timely intervention are essential to mitigate risks, prevent orbital compartment syndrome, and preserve vision.
